# “I don’t think I even thought of myself” A mixed-methods study of family experiences of trio germline whole genome sequencing in newly diagnosed childhood cancer

**DOI:** 10.1038/s41416-026-03354-9

**Published:** 2026-03-05

**Authors:** Jacqueline D. Hunter, Kate Hetherington, Maeve McGillycuddy, Claire E. Wakefield, Katherine M. Tucker, Tracey A. O’Brien, Brittany C. McGill, Noemi A. Fuentes-Bolanos, Kanika Bhatia, Bhavna Padhye, Andrew Grant, Kristine Barlow-Stewart, Meera Warby, Eliza K. Courtney, Michelle Peate

**Affiliations:** 1 Department of Obstetrics, Gynaecology and Newborn Health, Royal Women’s Hospital, Melbourne Medical School, Faculty of Medicine, Dentistry and Health Sciences, University of Melbourne, Victoria, Australia; 2 School of Clinical Medicine, Randwick Clinical Campus, Discipline of Paediatrics and Child Health, UNSW Sydney, Sydney, NSW Australia; 3 Behavioural Sciences Unit, Kids Cancer Centre, Sydney Children’s Hospital, Sydney, NSW Australia; 4 Stats Central, Mark Wain Wright Analytical Centre, UNSW Sydney, Sydney, NSW Australia; 5 Division of Quality of Life and Pediatric Palliative Care, Department of Pediatrics, Stanford University and Stanford Medicine Children’s Health, Palo Alto, CA USA; 6 Hereditary Cancer Centre, Department of Medical Oncology, Prince of Wales Hospital Randwick, Sydney, NSW Australia; 7 Prince of Wales Clinical School, UNSW Sydney, Sydney, NSW Australia; 8 Kids Cancer Centre, Sydney Children’s Hospital, Sydney, NSW Australia; 9 Children’s Cancer Institute, Lowy Cancer Research Centre, UNSW Sydney, Sydney, NSW Australia; 10 Children’s Cancer Centre, Royal Children’s Hospital Melbourne, Victoria, Australia; 11 Cancer Centre for Children, The Children’s Hospital at Westmead, Sydney, NSW Australia; 12 Kids Research, Children’s Cancer Research Unit, The Children’s Hospital at Westmead, Sydney, NSW Australia; 13 School of Nursing and Midwifery, Faculty of Health, University of Technology Sydney, Sydney, NSW Australia

**Keywords:** Ethics, Cancer genetics, Cancer genomics, Psychology, Paediatric cancer

## Abstract

**Background:**

Germline genomic sequencing (GGS) is increasingly offered to children with cancer. We explored families’ experiences of consent, result-disclosure, and satisfaction in the PREDICT study, a standalone trio-GGS study of unselected, newly-diagnosed patients ( ≤ 21 yrs).

**Methods:**

Using a convergent parallel mixed-methods design, parents and children ( ≥ 12 yrs) completed questionnaires at baseline/post-consent (T0), results-return (T1), and for parents, one-year post-enrolment (T2). Parents completed T1 interviews.

**Results:**

187/248 parents (mean:40.4 yrs) and 19/32 children (mean:14.9 yrs) from 128/144 families participated; 49 parents were interviewed. Few reported thoroughly reading consent materials and consent-related distress was low, though higher among parents with lower-income (*p* = 0.001) or below-average genetics knowledge (*p* = 0.027). At result-return, participants reported moderate distress, with no differences by result type (*p* = 0.118). Satisfaction was high (median: parents 98/100, children 87/100), 96% of parents and 60% of children would recommend PREDICT, and parents reported minimal regret (mean:15.74/100). Qualitative data revealed that cancer diagnosis-related distress influenced consent comprehension and potentially impeded parents’ ability to consider study implications for themselves. Emotional reactions to results ranged from relief to distress, regardless of findings. Communication and trust shaped experiences.

**Conclusions:**

Consent for trio-GGS at cancer diagnosis is complex, requiring flexible, tailored processes. Clear, timely communication from trusted clinicians is key to improving family experiences.

## Background

Between 10 and 18% of children with cancer carry a germline pathogenic or likely pathogenic variant (P/LPV) in a cancer predisposition gene, associated with a genetic cancer risk [1–4]. Benefits of identifying these variants are well established, including potential to clarify the cancer diagnosis, inform prognosis, and guide treatment, cancer surveillance, reproductive planning, and familial testing. Unlike phenotype-driven approaches, broad germline genomic sequencing (GGS) may identify germline P/LPVs in patients with no suggestive features or family history of cancer. This has led to widescale research-based rollout in multiple high-income countries [3–7].

Understanding families’ experiences is critical to inform integration of GGS for genetic cancer risk into routine clinical childhood cancer care. Previous studies exploring parent and child experiences of GGS for childhood cancer indicate high satisfaction with the testing process and outcomes [8–13]. Families have also previously described valuing the opportunity to provide feedback on their experiences with GGS, appreciating a holistic approach to their care [14]. However, with notable exceptions [9, 10, 12], most of these previous studies were conducted in the context of paired tumour-germline sequencing for selected patient populations embedded within precision medicine programs. Whilst precision medicine is expected to become standard practice for children diagnosed with cancer [15, 16], exploring family experiences in paired tumour-germline settings makes it difficult to distinguish outcomes specific to GGS. Reflecting this challenge, families have also described difficulty differentiating between germline and tumour sequencing when offered together [17].

In some cases, GGS is delivered as trio testing involving sequencing of parent-child pairs to inform variant filtering and inheritance patterns [18]. Whilst trio-based approaches can identify clinically relevant conditions such as parental mosaicism [19], they introduce emotional complexity for families. Uncovering parental genetic status may lead to guilt and anxiety for parents [12, 20, 21], which could be a potential barrier to their participation. We recently reported that healthcare professionals may be reluctant to enrol families in trio-GGS due to concerns about psychosocial challenges associated with GGS delivered at cancer diagnosis or as a trio-based approach [22]. Families have also reported that enrolment at cancer diagnosis can be overwhelming and reduce recall of consent discussions [8]. Given this lack of consensus and the potential risks of overwhelming families at diagnosis, the suitability of trio-based approaches in this setting should be considered.

Whilst studies exploring the psychological impact of receiving genomic information on children and their parents have so far identified few serious negative impacts [8, 23, 24], some evidence suggests identifying a P/LPV in a cancer predisposition gene is associated with increased distress [13, 25, 26]. Additionally, previous studies have found that families receiving GGS results for childhood cancer can struggle to recall their results [17, 27, 28]. Some have inaccurately described receiving a germline P/LPV result when they did not [17], whereas others, including adolescent and young adult patients, have reported being unable to accurately recall receiving a reportable finding [27, 28]. These studies have called for further research exploring families’ preferences for return of GGS results to inform processes to improve recall and understanding [27, 28].

Most previous studies on GGS in paediatric oncology have been conducted in a precision medicine context, with patients selected for specific tumour types. These studies have not generated consensus regarding the ideal time to offer testing, or the acceptability and psychosocial impact of trio-based approaches at cancer diagnosis. Additionally, ongoing research on families’ experiences is needed to adapt genomic testing as new evidence emerges in this rapidly evolving field. Ongoing research will also help identify families at higher risk of distress, informing strategies to support them. This, in turn, can reassure clinicians who may be reluctant to enrol families in GGS for genetic cancer risk due to psychosocial concerns [22].

To meet this research need, we conducted a mixed-methods study to explore families’ experiences of a standalone trio-GGS study for genetic cancer risk, not paired with tumour testing, in a cohort of unselected, newly-diagnosed children with cancer.

### Research questions

1. What are parents’ and children’s experiences of the consent process for trio-GGS, do they find it distressing, and which factors, if any, influence this distress?
2. What are parents’ and children’s experiences of receiving trio-GGS results, do they find result disclosure distressing, and which factors, if any, influence this distress?
3. How satisfied are participants, does this change over time, and does the receipt of trio-GGS results influence satisfaction?

## Methods

The detailed study protocol has been published [29].

### Participants and Study Design

#### The PREDICT study

The Cancer PREDisposItion in Childhood by Trio-based sequencing (PREDICT) Study was an Australian state-based (NSW) multicentre GGS study. All consecutive newly-diagnosed paediatric and adolescent patients with cancer ( ≤ 21 years, referred to hereafter as ‘children’) at participating sites during the study period were offered enrolment and consent by their treating clinician, regardless of clinical suspicion, cancer type, or family history of cancer. Children and both their biological parents (where possible) provided blood samples for trio-GGS analysis. Multidisciplinary teams assessed sequencing and variant curation data and issued results reports to treating clinicians indicating no reportable findings or detailing reportable findings and recommendations based on findings. Reportable findings included P/LPVs in a curated list of cancer predisposition genes, regardless of zygosity and inheritance [29]. All findings in these genes were reported, even if not cognisant with the child’s cancer or in adult-onset cancer predisposition genes. The child’s treating clinician then disclosed results to families and determined next steps, including referral to clinical genetics, where appropriate, based on each family’s clinical situation. All families with reportable findings were recommended clinical genetics referral. Some families whose results did not identify a P/LPV received a recommendation for a clinical genetics referral in their PREDICT results based on additional information identified during the study including relevant family cancer history, diagnosis, or suspicious clinical features.

#### The PREDICT-Impact study

The PREDICT study included a prospective longitudinal mixed-methods psychosocial component, known as the PREDICT-Impact study. Both parents and/or guardians (referred to hereafter as ‘parents’) of enrolled children and children ( ≥ 12 years) were eligible to participate in PREDICT-Impact. All families enrolled on PREDICT were also enrolled in PREDICT-Impact on an opt-out basis. A trained psychosocial researcher (JDH) then contacted parents to conduct an intake call confirming their participation, with families again able to opt-out of PREDICT-Impact at this time. After confirming their participation, parents were invited to complete questionnaires and a semi-structured interview, while children were invited to complete questionnaires only. We used a convergent parallel mixed-methods study design, analysing quantitative and qualitative data independently, before comparing side by side and identifying overlapping themes and concepts to triangulate results [30].

### Data collection

We sent parents and children online questionnaires after consent and study enrolment (Time 0, T0) and after the return of their PREDICT trio-GGS results (T1). Parents additionally completed questionnaires one year after study enrolment (T2) and an optional semi-structured interview at T1.

Two researchers (KBS, JDH) conducted audio-recorded interviews via telephone, transcribing verbatim. Guided by the principles of information power [31], the primary interviewer (KBS) deemed that after 34 interviews, few new insights were emerging for parents who received no reportable findings, whilst the perspectives of families who received reportable findings or a recommended clinical genetics referral were underrepresented. We continued purposive sampling of these parents to enhance the diversity and depth of experiences captured in the qualitative analysis.

### Measures

A multi-disciplinary expert panel comprising paediatric oncologists, genetic counsellors, clinical geneticists, psychologists, and psychosocial researchers developed and pilot tested questionnaires and interview guides. The study team collected and stored basic demographics, clinical information, and trio-GGS results in the PREDICT study database. We extracted relevant parent and child data from these records (Supplementary Table 1). Parents provided further demographic information in T0 questionnaires. Questionnaires also included validated, purpose-developed, and adapted items from previous research exploring participants’ perspectives towards PREDICT consent information [32], purpose-developed distress scales (where 1=low, 10=high distress) related to the consent process, such as deciding to provide a sample of their child’s genetic material for trio-GGS (referred to as ‘consent-related’ distress), and receipt of trio-GGS results (referred to as ‘result-related’ distress), satisfaction [33], willingness to recommend PREDICT to others [33], and the Decisional Regret Scale [34] (Supplementary Table 1). Child questionnaires included the same domains as parents, with items adapted by the multidisciplinary panel to include fewer or simpler questions and more accessible language to ensure a Flesch-Kincaid readability of grade 5.0 (Supplementary Table 2). Questionnaires included free-text response boxes to allow participants to provide additional information.

Parents’ semi-structured interviews explored experiences of consent, result return, and overall study satisfaction (Supplementary Table 3).

### Data analysis

#### Quantitative data

We analysed quantitative data using SPSS (v30.0.0.0) [35] and R (v4.1.2) [36]. We used a significance threshold of *α* = 0.05 for all statistical tests. Predictors were tested using F-tests in linear mixed models and likelihood ratio tests in non-Gaussian mixed models. Wald confidence intervals and p-values are reported for parameter effect estimates. Model assumptions were assessed by examining residual plots, checking the proportional odds assumption for ordinal models, and estimating the variance inflation factor to assess multicollinearity.

##### Consent-related and result-related distress

To identify factors associated with parental consent-related and result-related distress, we fitted mixed effects models to account for clustering within families. Based on the distribution of responses, we used ordinal mixed effects regression for consent-related distress and linear mixed effects regression for result-related distress. Predictors were selected based on previous literature and expert consensus and included self-reported genetics knowledge, relationship status, annual household income, having other children, family history of cancer, cancer diagnosis, and parent gender [8, 17, 25]. For consent-related distress, we included time since cancer diagnosis to enrolment as an additional predictor (Supplementary Table 4). For result-related distress, we included result type and time since consent to receipt of results as additional predictors (Supplementary Table 5). Missing data ranged from 9 to 14% across variables included in these models. A complete case analysis was conducted under the assumption that data were missing at random. Multiple imputation analyses were performed using the mice algorithm [37] with 50 imputations to assess the robustness of the findings.

##### Overall satisfaction

To assess differences in participants’ willingness to recommend the study over time (T0–T1/T2), we used Fisher’s Exact Test. We estimated parent satisfaction at T1/T2 using generalized linear mixed models (GLMMs) fitted to the satisfaction deficit (scores < 100), assuming a Tweedie distribution via the glmmTMB package [38], with nested random intercepts for parent and family and adjustment for baseline satisfaction (T0). At T1, we examined associations between satisfaction and result type and time taken to receive results. At T2, we examined the association between receipt of result and satisfaction. The model provides unbiased estimates when data is missing at random for T1 and T2 non-responders. Sensitivity analyses were conducted by fitting models to complete cases.

#### Qualitative data

Using Nvivo (QSR International Pty Ltd. Version 14, 2024), we conducted a reflexive thematic analysis of qualitative interview and open-ended questionnaire response data within a pragmatic-constructionist epistemological framework [39, 40]. One psychosocial researcher with postgraduate training in qualitative research methodology (JDH) familiarised herself with the data by reviewing responses and transcripts against the original audio. JDH then conducted an initial round of coding using the research aims as a structural framework to organise the data. JDH then engaged in inductive, iterative, and reflexive coding to develop interpretive codes summarising points of meaning. JDH collaboratively discussed codes and subcodes with another psychosocial researcher with postgraduate training in psychology and qualitative research methods (KH) until they identified patterns of meaning underlying the data representing themes and subthemes. Two researchers (JDH, KH) then worked together to refine and name themes representing interpretive constructs derived from the data.

## Results

### Participants

Overall, 187 parents (mean age: 40.4 years, 62% female/mothers) and 19 children (mean age:14.9 years, 68% female) from 128 families completed T0 (representing 17 dyads); 49 parents were interviewed. The average time between cancer diagnosis to T0 completion was 109.63 days for parents (SD = 67.68) and 112.79 for patients (SD = 75.90). Of the 128 participating families, 24 (19%) had a child who received reportable findings and referral to clinical genetics, 30 (23%) did not have reportable findings but received a recommendation for clinical genetics referral, 69 (54%) had no reportable findings or referral recommendation, and 5 (4%) did not provide a sample and therefore did not proceed with GGS after consent, participating only at T0. Table 1 provides demographic and clinical features of participating families, while Supplementary Table 6 provides demographic characteristics of participating parents.

**Table 1:** Demographic and clinical characteristics of children of parents participating in PREDICT-Impact questionnaires, children of parents participating in PREDICT-Impact Interviews, and children participating in PREDICT-Impact for themselves.

	Children of parents participating in PREDICT-Impact questionnaires (*N* = 126)	Children of parents participating in PREDICT-Impact interviews (*N* = 42)	Children participating in PREDICT-Impact questionnaires (*N* = 19)
**Age at enrolment in PREDICT, years**			
Mean (SD)	7.6 (4.9)	7.6 (5.2)	14.9 (1.5)
Range	0.2–17.0	0.2–16.5	12.1–17.5
**Age at diagnosis, years**			
Mean (SD)	7.5 (5.0)	7.5 (5.2)	14.8 (1.6)
Range	0.2–16.9	0.2–16.5	11.9–17.3
**Time from diagnosis to enrolment, days**			
Mean (SD)	60.8 (55.8)	58.2 (43.4)	44.7 (25.9)
Range	0–432	8–230	3–84
**Sex, no. (%)**			
Female	52 (41.3)	19 (45.2)	13 (68.4)
Male	74 (58.7)	23 (54.8)	6 (31.6)
**Diagnosis, no. (%)**			
Central nervous system tumours	12 (9.5)	7 (16.7)	4 (21.1)
Non-CNS solid tumours			
Sarcoma	16 (12.6)	6 (14.3)	1 (5.3)
Neuroblastoma	6 (4.8)	1 (2.4)	-
Other solid tumour	23 (18.3)	11 (26.2)	4 (21.1)
Haematological malignancies	69 (54.8)	17 (40.5)	10 (52.6)
**Risk stratification**^a^			
Low/standard	28 (22.2)	5 (11.9)	4 (21.1)
Medium/intermediate	17 (13.5)	4 (9.5)	3 (15.8)
High	19 (15.1)	8 (19.0)	3 (15.8)
Unknown at time of enrolment	30 (23.8)	7 (16.7)	4 (21.1)
Not applicable^b^	23 (18.3)	16 (38.1)	4 (21.1)
(missing)	9 (7.1)	2 (4.8)	1 (5.3)
**Aboriginal or Torres Strait Islander, no. (%)**			
Yes	9 (7.1)	-	-
No	116 (92.1)	42 (100)	19 (100)
(missing)	1 (0.8)	-	-
**Cultural background**^**c**^**, no. (%)**			
Australian	105 (83.4)	39 (92.9)	15 (79.0)
Western/European	4 (3.2)	-	-
Non-Western/European	14 (11.1)	3 (7.1)	4 (21.1)
(missing)	3 (2.4)	-	-
**First language, no. (%)**			
English	114 (90.5)	37 (88.1)	19 (100)
Other	3 (2.4)	-	-
(missing)	9 (7.1)	5 (11.9)	-
**Rurality, no. (%)**			
Capital city	80 (63.5)	27 (64.3)	12 (63.2)
Other metropolitan centre	17 (13.5)	6 (14.3)	3 (15.8)
Rural and remote areas	28 (22.2)	8 (19.0)	4 (21.1)
(missing)	1 (0.8)	1 (2.4)	-
**Family history of cancer, no. (%)**			
Collected, informative	13 (10.3)	8 (19.0)	3 (15.8)
Collected, not informative	65 (51.6)	21 (50.0)	11 (57.9)
Not collected	44 (34.9)	13 (31.0)	4 (21.1)
(missing)	4 (3.2)	-	1 (5.3)
**GGS result, no. (%)**			
Reportable findings	24 (19.0)	16 (38.1)	1 (5.3)
Child-onset CPG	8 (6.3)^d^	5 (11.9)^f^	
Adult-onset CPG	5 (4.0)^e^	3 (7.1)^g^	
Carrier for recessive CPG	11 (8.7)	8 (19.0)	1 (100.0)
Clinical genetics referral recommended	29 (23.0)	13 (31.0)	7 (36.8)
No reportable findings or referral	69 (54.8)	13 (31.0)	10 (52.6)
No report issued – patient samples not obtained	4 (3.2)	-	1 (5.3)
**Time to result if received, days**	*n* = 95	*n* = 42	*n* = 15
Mean (SD)	265.0 (94.1)	243.2 (80.2)	291.2 (96.1)
Range	92–493	92–386	154–489
(missing)	6	1	-

*CPG* cancer predisposition gene.

*GGS* germline genomic sequencing.

^a^Risk stratification based on Children’s Oncology Group Risk Stratification Guidelines.

^b^Diagnoses with no standardised risk stratification information available.

^c^Those who described their cultural background as Australian and another culture were classified as ‘Australian’. Those who described their cultural background as Western/European and another culture were classified as ‘Western/European’.

^d^1 child in this category also had a carrier finding in a recessive CPG.

^e^2 children in this category also had a carrier finding in a recessive CPG, 1 had a finding in 2 different adult-onset CPGs.

^f^1 child in this category also had a carrier finding in a recessive CPG.

^g^1 child in this category also had a carrier finding in a recessive CPG, 1 had a finding in 2 different adult-onset CPGs.

**Fig. 1:**
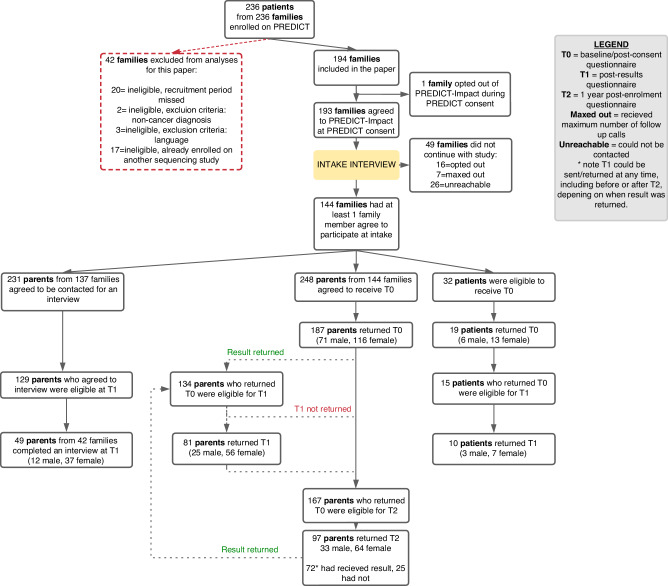
Recruitment flow chart. Of 194 families included in this paper, 144 had at least one family member agree to participate in PREDICT-Impact, including 248 parents and 32 children (74% family response rate). 187 parents (187/248, 75% parent participation rate) and 19 children (19/32, 59% child participation rate) from 128 families (128/144, 89% family participation rate) returned T0. 231 parents opted for an interview at study intake, 129 of which were eligible at T1. Of those, 49 completed interviews (38% interview participation rate). Using a logistic regression model, we did not identify any strong evidence to suggest that the child’s cancer diagnosis, age at enrolment, geographic location, cultural background, preferred language, or recruiting hospital significantly influenced the likelihood of family participation in PREDICT-Impact (*p* > 0.05). T1 could occur before or after T2, depending on when the PREDICT results were made available to the family, which at times was more than one year after enrolment. Participants were only eligible to receive T1 if the treating clinician provided confirmation to the study team that PREDICT results had been returned to the family. At times families received their results but this information was not shared with the study team. In these cases, parents may have completed T2 after receiving their result, despite not yet being sent T1. These families became ineligible to receive T1.

Of these participants, 81 parents and 10 children also completed the T1 questionnaire, and 97 parents completed the T2 questionnaire, 72 of whom (74%) had received their PREDICT results at the time of completion. Figure 1 summarizes overall study response and participation and the flow of participants through each study timepoint.

### Consent experiences

#### Perspectives towards consent information

At T0, most children engaged with the Participant Information and Consent Form (PICF) information ‘briefly’ (11/19, 58%) or ‘not at all’ (6/19, 32%). Only one child read the information ‘quite thoroughly,’ and one read ‘just the parts they felt were relevant.’ Most who read the information thought the length was ‘just right’ (8/13, 62%).

Parents read the PICF ‘from cover to cover’ (40/181, 22%), ‘quite thoroughly’ (46/181, 25%), ‘briefly’ (48/181, 27%) or ‘just the parts that were relevant’ (38/181, 21%). Few did not read the information at all (9/181, 5%). Most parents who engaged with the PICF thought the length was ‘just right’ (150/170, 88%), with few finding it ‘too long’ (17/170, 10%) or ‘too short’ (3/170, 2%).

#### Consent-related distress

At T0, most children reported minimal worry due to their participation in PREDICT, with 16/19 children (84%) indicating they felt ‘not at all’ worried. Three expressed at least ‘a little’ worry (3/19, 15%). Half of children (10/19, 53%) reported feeling at least ‘a little’ happy or reassured because of their participation in PREDICT, while the remainder reported that PREDICT did not make them feel happy or reassured (9/19, 47%).

**Fig. 2:**
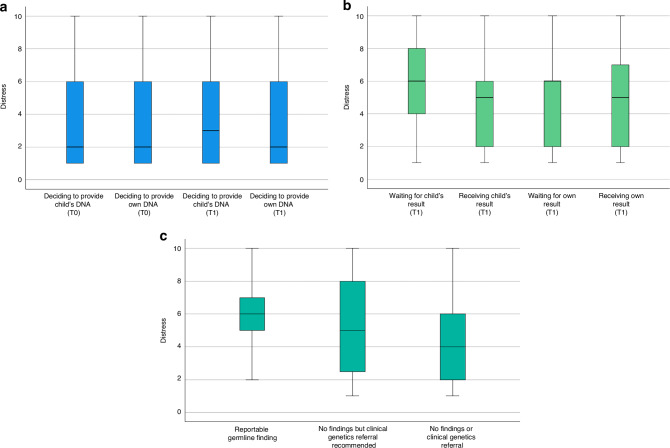
Box and whisker plots of parent consent-related and result-related distress ratings. **a** At T0 and T1, deciding to provide a sample of their child’s genetic material (*n*(T0) = 185; *n*(T1) = 78) or their own genetic material (*n*(T0) = 175; *n*(T1) = 64) to look for variants in cancer predisposition genes. **b** At T1, waiting for their child’s (*n* = 79) or their own (*n* = 63) PREDICT results and receiving their child’s (*n* = 74) or their own (*n* = 49) PREDICT results. **c** At T1, receiving their child’s PREDICT results, stratified by the type of result received (*n*(reportable germline finding) = 17, *n*(no finding but clinical genetics referral recommended) = 12, *n*(no findings or referral) = 45). Ratings from 1 to 10 where 1 is ‘not at all distressing’ and 10 is ‘extremely distressing’. T0 = baseline/post-consent questionnaire, T1 = post-results questionnaire.

At T0 and T1, parents rated their child’s cancer diagnosis as highly distressing (T0: median = 10, IQR = 1; T1: median = 10, IQR = 1). At T0, 124/186 (67%) gave the maximum possible distress rating of 10. Distress ratings were low regarding the decision to provide a sample of their child’s genetic material (median = 2, IQR = 5) or their own genetic material (median = 2, IQR = 5) for GGS at T0, remaining low at T1 (Fig. 2a).

In regression analysis examining parents’ distress regarding deciding to provide a sample of their child’s genetic material for GGS (T0) we found strong evidence of an association with annual household income ($${\chi }_{2}^{2}$$ = 11.95, *p* = 0.003) and genetics knowledge ($${\chi }_{1}^{2}$$ = 4.89, *p* = 0.027)(Supplementary Table 4). Parents with an income above $120,000 reported lower consent-related distress compared to parents earning less than $120,000 (OR = 0.23, 95% CI = [0.09, 0.57], *p* = 0.001). Parents with below-average genetics knowledge reported higher consent-related distress compared to those with average or above-average knowledge (OR = 2.29, 95% CI = [1.09, 4.76], *p* = 0.027). There was also strong evidence of an association between genetics knowledge and parent distress regarding providing their own genetic material at T0 ($${\chi }_{1}^{2}$$ = 6.54, *p* = 0.011), with parents with below average genetics knowledge again reporting higher consent-related distress (OR = 3.22, 95% CI = [1.31, 7.90], *p* = 0.011). We found no evidence of an association with time since cancer diagnosis, having other children, family history of cancer, cancer diagnosis, marital status, or gender (Supplementary Table 4). Findings were consistent with multiple imputation analyses (results not shown).

#### Qualitative themes characterising consent experiences

Qualitative exploration of families’ experiences with the PREDICT consent process revealed themes related to genetic responsibility, trust, altruism, and navigating study demands (Table 2).

**Table 2:** Qualitative themes and sub-themes with representative quotes from parents and children.

Themes and subthemes		Parent	Child
**Consent experiences**			
Carrying the burden of genetic responsibility at diagnosis	Adjusting to the emotional shock of cancer diagnosis	“The extent of the trauma and shock, it’s like we were being sucked through a conveyer belt of processes and, and I just don’t remember who said what to me.” (mother, 48, child with CNS tumour) “When it starts, it’s the worst time of your life… you can’t see the forest for the trees.” (mother, 51, child with non-CNS solid tumour) “We had enough time under our belt to know what we were dealing with… at the start, you don’t even know what to expect. So maybe we were better armed to make a decision at the time that we got told about it cause we’ve been through one or two cycles of chemo.” (mother, 45, child with non-CNS solid tumour)“	“It didn’t make a big impact, too much was going on for something like this to really matter.” (child, 15, haematological malignancy) “It wasn’t something I spent much time thinking about to be honest since I had only just found out I had cancer when I was asked to take part.” (child, 13, non-CNS solid tumour)
	Confronting guilt and genetic blame	“This is really actually quite scary. What if we find out something we don’t wanna know?” (mother, 34, child with non-CNS solid tumour) “I was happy to provide my child’s DNA for testing, since the benefit to her was clear. However, choosing supply my own DNA for predisposition testing was a longer decision. The notion that either parent could be identified as genetically ‘responsible’ for our daughter’s cancer is an emotionally and psychologically confronting possibility.” (father, 35, child with haematological malignancy)	“It scared me to think that there is something inside me or my family that could affect my future family or my brothers.” (child, 13, haematological malignancy) “It may put extra stress on my parents and myself, and I worry my results will not be good.” (child, 16, haematological malignancy)
Trusting the system to cope with overwhelm	Trusting the clinician	“It’s too hard to work out… you just put all your trust in everyone around you.” (mother, 51, child with non-CNS solid tumour) “We really trusted [name of doctor]… she’s the genetic doctor] and she’s very connected to the research. So it didn’t feel like it was a strange person coming in to ask us, it felt like someone who knew [name of daughter] and knew us.” (mother, 37, child with non-CNS solid tumour)	“Someone came and talked about this study [and] explained [it] well … I was happy to be part of this study.” (child, 13, haematological malignancy)
	Trusting the healthcare system	“I’ve worked for many years in research… I know it’s a very good way to access certain healthcare or testing and things that would not otherwise be available to you.” (mother, 38, child with non-CNS solid tumour) “I trusted the system… the environment in Australia and the medical system in Australia definitely gave us trust from the start.” (father, 34, child with non-CNS solid tumour)	
	When trust feels undermined	“There wasn’t any, you know, how are you going? Just sign up to this study so we can all get some information that’s gonna make us look amazing.” (mother, 51, child with non-CNS solid tumour) “I think that it’s unfortunate insurance is a factor people need to consider to support cancer children research” (father, age missing, child with haematological malignancy)	
Altruism amid vulnerability	Desire to help family	“Information is power. So, if there’s stuff that we need to know that can affect how closely we monitor [child] or what risk he’s at in the future, then, whilst it’s really confronting, it’s better to know it.” (mother, 45, child with non-CNS solid tumour)	“I am grateful that my health and wellbeing are being cared for and a treatment plan has been devised as this will give me the best chance of being cured.” (child, 16, haematological malignancy)
	Desire to help others	“Any research … whether it’s little or a lot, will always help in future. So we were more than happy to participate in the study.” (mother, 41 child with non-CNS solid tumour) “I just want to help as much as I can, any other family in the future that come across the same sort of thing… So, we, I signed all the paperwork, to be honest with you. I pretty much didn’t read anything.” (mother, 33, child with non-CNS solid tumour)	“I don’t really know what the study is about but if it can help future kids in my condition then I’ll gladly help.” (child, 15, haematological malignancy)
	Prioritising others over yourself	“I don’t think I even thought about myself.” (mother, 48, child with CNS tumour) “I never worried about what I might have because I just kept wishing it was me, not her, at any point. So I wasn’t thinking, oh, I hope they don’t find cancer in me.” (mother, 51, child with non-CNS solid tumour)	“Didn’t want to participate, but mum and dad explained we could be helping others and our family with the genetic testing.” (child, 14, CNS tumour)
Navigating study demands during difficult times	Logistical study demands	“To be honest, I haven’t had my blood tests yet because when she was going through radiation, we were at Ronald McDonald House and you needed one parent with your child at the hospital and one parent with our kids at Ronald McDonald House…so there was never any time to get the test done.” (mother, age missing, child with CNS tumour)	
	Emotional study demands	“I should say that I’m the worst child in the whole world, and I have a particular phobia of needles. So I hate going to have blood taken for anything. Um, and this was no exception.” (father, 48, child with CNS tumour)	“I was ok to participate as long as I don’t need to get a needle.” (child, 12, CNS tumour)
**Result-return experiences**			
Emotional meaning-making	Relief and reassurance: no findings	“It was a relief that it wasn’t from myself or [partner]… that it was not our fault…It just takes the sort of the guilt away.” (mother, 41, child with CNS tumour) “It would be really distressing in a different context. But nothing can be worse than your kid having cancer. And so it was actually reassuring to have this option to understand more. Obviously this is coming from the perspective of someone who received good news that no gene faults were found.” (mother, 35, child with haematological malignancy)	“I was happy that I don’t have any mutations that may cause cancer in my offspring.” (child, 17, haematological malignancy) “Receiving the results was a big relief.” (child, 14, haematological malignancy)
	Relief and reassurance: reportable findings	“The good news is although there was a genetic abnormality that was found in both [child] and [partner], it’s not one that is linked to osteo. So, you know, we got a good outcome from this hopefully we’ve actually now got the reassurance that he doesn’t have that. So that in itself is a benefit because it reduces anxiety.” (mother, 45, child with non-CNS solid tumour, reportable finding: child-onset CPG)	
	Disappointment: no findings	“Oh, I think it’s quite disappointing…It still doesn’t provide us with clear answers…. I wish I did have a conclusive answer to why this has happened to her.” (mother, 46, child with CNS tumour)	
	Disappointment: reportable findings	“I was disappointed. I wasn’t surprised. It was just disappointing. I was just hoping that she had already been dealt her unlucky cancer genes that she wouldn’t have to worry about it. I was hopeful, but that didn’t matter. She’s got it.” (mother, 35, child with haematological malignancy, reportable finding: adult-onset CPG)	
	Distress: no findings	“There’s no reason for my genes and her father’s genes to have contributed to this… It’s just a fluke. Mind you, it has to do with, with her genetics, it has to do with me… I don’t want to say to her father, but I am blaming myself for this because had I taken care of myself better when I was pregnant with her, she probably wouldn’t have been going through what she’s going through.” (mother, 41, child with non-CNS solid tumour)	
	Distress: reportable findings	“A blood sample showed that it came from me…I was a little bit upset about it a little bit, you know, I guess guilty, anxious, that kind of thing.” (mother, 44, child with non-CNS solid tumour, reportable finding: carrier finding in recessive CPG) “I did have a little cry and I felt quite shocked about the news.” (mother, 45, child with non-CNS solid tumour, reportable finding: child-onset CPG)	
	Distress: waiting for results	“There’s been such a long wait for these results and I know I was really anxious at times.” (mother, 43, child with haematological malignancy, reportable finding: carrier finding in recessive CPG)	“Just the long wait for the results, I thought that they found something and they were looking into it more before returning to us.” (child, 14, haematological malignancy)
	Indifference: no findings	“With everything else that’s been going on, this seemed somewhat pedestrian…I’d actually forgotten about the whole study. It came so long after. And because it was, you know, a non-event, I was just like, yep, okay. And carried on and didn’t give it anymore thought. Had it come back differently then I may well have reacted differently.” (father, 48, child with CNS tumour)	
	Indifference: reportable findings	“Honestly, I didn’t really react or feel anything…. I don’t know if I’ve switched off that emotion…I mean, worst case happened, he was diagnosed with cancer. So whether he’s got the gene, he doesn’t have the gene. I actually don’t understand what that means.” (mother, age missing, child with non-CNS solid tumour, reportable finding: child-onset CPG)	
	Indifference: waiting for results	“To be honest, I think we’ve had so much happening, it sort of went on the back burner in terms of thinking about it.” (father, 55, child with haematological malignancy)	
Balancing trust and expertise	Trust in familiarity	“He was very upfront about the fact that this wasn’t his area of expertise…but he sort of gave me the top line figure. The thing that I’ve always really appreciated about [oncologist] is that I trust him completely…I felt like I could trust what he was saying.” (mother, 45, child with non-CNS solid tumour, reportable finding: child-onset CPG) “I remember it was the same oncologist who actually told us about… how all of this works. And she’s very lovely. So it was very nice of her. She handed it in herself and, you know, reassured me there is no bad genes from me.” (mother, 36, child with non-CNS solid tumour)	
	Trust tested by limited expertise	“It would’ve been nice to be taken through all of that in much more detail.” (mother, age missing, child with non-CNS solid tumour, reportable finding: child-onset CPG) “No, I don’t, I dunno if, I don’t think I did [get results]. I got a lot of paperwork. It might be here somewhere, I’m not sure, but I don’t think so.” (father, age missing, child with non-CNS solid tumour) Interviewer: “Did you feel comfortable asking questions about the results?” Parent: “Not really. With her, she was very clear that she was not an expert in this situation.” (mother, 35, child with haematological malignancy, reportable finding: adult-onset CPG)	
**Overall satisfaction**			
**Satisfied because…**			
Finding purpose and personal benefit	Making an altruistic contribution	“If they found something I would’ve definitely told my sisters and, you know, they have got kids as well.” (father, 50, child with haematological malignancy) “If it helped anybody or it, um, kind of helped research, it gave us any information than it was worthwhile to do. And even if it just helped with research.” (mother, 48, child with CNS tumour)	**“**I was pleased to be able to participate and help future generations.” (child, 16, haematological malignancy)
	Emotional relief and empowerment	“I feel like it was totally beneficial… the more knowledge the better. I would rather know, even if it’s bad news, I’d rather know what we’re dealing with and be able to be prepared.” (father, 41, child with non-CNS solid tumour, reportable finding: carrier finding in recessive CPG)	
**Dissatisfied because…**			
Compromised trust in the system and process	Poor communication eroding trust	“It has been distressing not getting any results of tests that were done.” (mother, 45, child with haematological malignancy) “We gave blood more than a year ago now and nobody has been in contact with results. Surely a short email with the results isn’t too much to ask.” (father, age missing, child with non-CNS solid tumour) “I know initially if there were questions there was never really anyone to contact.” (father, 41, child with non-CNS solid tumour) “Communication throughout the whole process has been poor. We have received no support.” (father, age missing, child with non-CNS solid tumour)	
	Perceived lack of procedural integrity	“We have concerns over how personal medical information is stored and how secure this will be. i.e. sold at a later date or extended to form part of another study that may be accessible to others.” (father, 39, child with haematological malignancy) “I think there’s logistical reasons as to why this happens… But the genetics team was so, um…practical, comforting, realistic… if that could have happened sooner after you sort of hear the slightly bad news that there is something there… that maybe could have helped.” (mother, 45, child with non-CNS solid tumour, reportable finding: child-onset CPG)	

*CNS* central nervous system.

*CPG* cancer predisposition gene.

#### Carrying the burden of genetic responsibility at diagnosis

Parents and children described how their engagement with consent information often depended on their adjustment to the emotional shock of the recent cancer diagnosis, a traumatic time characterised by ‘information overload’. Whilst negotiating this emotional overload, some shared that “the extent of trauma and shock” (mother, 48 years) they were experiencing made it difficult to process or recall study details. Others felt they had “enough time under [their] belt” (mother, 45 years) to adjust to the diagnosis and engage with information or emphasized that gaining experience through starting treatment and having a plan in place helped them to feel better prepared to absorb information. Some parents and children shared that the consent process itself was distressing as it meant confronting the challenges of genetic blame and guilt. Parents feared being “genetically responsible” (father, 35 years) for their child’s cancer, while children worried about their future and the impact on their family. Some parents reported that this emotional burden made it difficult for them to decide whether to provide their own sample for trio testing.

#### Trusting the system to cope with overwhelm

Parents and children described that engagement with the PREDICT consent process or materials was shaped by deep trust in the system i.e., the healthcare system and recruiting clinicians. This trust at times served as a vehicle for coping with emotional overwhelm: “I skimmed through it and just trusted that the doctors advising me [were] right” (mother, 52 years). However, when communication by recruiting clinicians was perceived as poor or self-serving, this trust was undermined, leading to negative feelings: “There wasn’t any ‘how are you going’ - just ‘sign up to this study so we…look amazing’” (mother, 51 years).

#### Altruism amid vulnerability

Parents and children also described a strong desire to gain benefits, whether for themselves, their family, or others, through contributing to research, even while grappling with their own distress. These motivations sometimes outweighed potential downsides of participation. For parents, participation centred on helping their child above all else, giving little thought to implications of participation on themselves. Children too described participating to help others, despite initial fears: “didn’t want to participate, but mum explained we could be helping others” (child, 14 years).

#### Navigating study demands during difficult times

Despite their altruism, participants faced practical challenges during the PREDICT consent process related to the demands of enrolment. These included logistical difficulties with blood sample collection and managing needle phobia, adding to emotional and logistical demands of PREDICT participation.

### Result experiences

#### Result return-related distress

At T1, 3/10 (30%) of children reported feeling at least ‘a little’ worried because of participating in PREDICT, while the remaining 7/10 (70%) indicated they were ‘not at all’ worried. Most children (6/10, 60%) reported feeling at least ‘a little’ happy or reassured by their involvement in PREDICT, with the remainder (4/10, 40%) indicating they were ‘not at all’ happy or reassured by their participation.

At T1, parents’ result-related distress ratings (Fig. 2) were moderate on average related to waiting for and receiving their child’s PREDICT results (waiting: mean = 5.62, SD = 2.72; receiving: mean = 4.76, SD = 2.66). For parents who indicated they provided a sample of their own genetic material for GGS, result-related distress ratings were also moderate on average (waiting: mean = 4.68, SD = 2.99; receiving: mean = 4.80, SD = 2.98).

Parents whose child received a reportable finding reported an average result-related distress score of 5.82 (SD = 2.01). Those who received no findings but a recommendation for clinical genetics referral reported a mean of 5.25 (SD = 3.19), and those who received no findings and no referral reported a mean of 4.22 (SD = 2.63). However, we found no evidence of an association between result-related distress regarding receiving their child’s result and GGS result type (F(2, 54) = 2.23, *p* = 0.118) or other key factors (Supplementary Table 5). Findings were consistent with multiple imputation analyses (results not shown).

#### Qualitative themes characterising result-return

Result experiences were characterised by qualitative themes related to the emotional weight of results and the impact of trust on experiences of the result return process (Table 2).

#### Emotional meaning-making

Families’ experiences of receiving GGS results were shaped not just by the outcome but also by how findings intersected with their ongoing cancer experience. The waiting period intensified feelings of uncertainty and fears of familial implications for some parents, with one father describing it as “quite a distressing wait” (father, 36 years). Others, primarily those who received no reportable findings, described the wait for results as non-distressing, with the entire process overshadowed by more immediate concerns related to the cancer.

Receiving results elicited diverse and meaningful emotional responses. For many, receiving results was “a big relief” (child, 14 years), particularly when no reportable findings were identified which alleviated parental guilt and provided comfort. Some without reportable findings described indifference to receiving results, citing other more important priorities. In cases where reportable findings were returned, some parents shared that they felt empowered by gaining more information, even if the germline finding was unlikely to be the cause of their child’s cancer. Others described guilt and shock, particularly if the finding was inherited from them. Even when no genetic cause was found, some parents shared that they still blamed themselves for their child’s cancer. Disappointment was described by participants who did not receive a reportable finding, leaving them without “clear answers” (mother, 46 years) explaining the cancer, and those who did, suggesting an ongoing genetic cancer risk.

#### Balancing trust and expertise

As with the consent process, experiences of receiving results were closely tied to the trust placed in clinicians and the broader health system. Trust functioned as a source of reassurance and a lens for interpretation of results. As one mother reflected, receiving results from her primary oncologist provided emotional security through familiarity: “I trust [them] completely” (mother, 45 yrs), despite genetics not being their area of expertise. Conversely, when communication felt fragmented or insufficient, trust was strained, leaving families with unanswered questions and heightened uncertainty. For example, some parents who received results from their oncology clinician rather than a genetics professional described being left with unanswered questions because they “were not an expert in this situation” (mother, 35 years).

### Overall study satisfaction

#### Satisfaction with participation

**Fig. 3:**
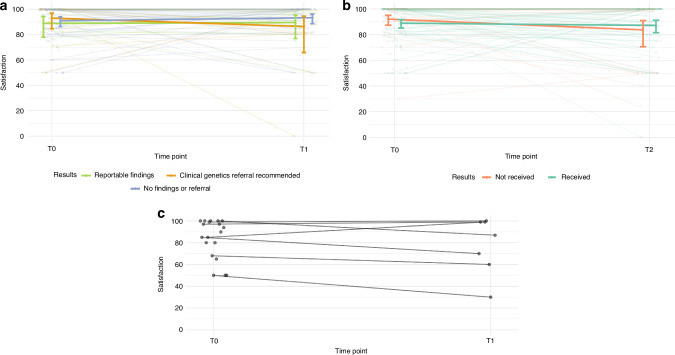
Parents’ satisfaction with participation in PREDICT. **a** Baseline/post-consent (T0, *n* = 182) to post-results (T1, *n* = 79) by the type of result received; **b** baseline/post-consent (T0) to 1-year post-enrolment (T2, *n* = 95) by whether they had received their PREDICT results by T2; and **c** children’s satisfaction with participation in PREDICT from T0 (*n* = 19) to T1 (*n* = 7). We estimated the marginal means (95% confidence intervals) of parents’ satisfaction with participation by result type from a Tweedie mixed-effects regression model.

At T0, children rated their overall satisfaction with their participation in PREDICT as high (median = 90, IQR = 32, *n* = 19). This remained high in those who received their result and completed T1 (median = 87, IQR = 39, *n* = 7)(Fig. 3c).

Parent satisfaction was high at T0 (median = 100, IQR = 17), with 52% of respondents (94/182) giving the maximum rating of 100. At T1, parents reported a median satisfaction score of 98 (IQR = 14, *n* = 79), and at T2, the median was 100 (IQR = 22, *n* = 95).

There was no evidence of a change in parent satisfaction ratings from T0 to T1 (${\chi }_{4}^{2}\,$= 3.8, *p* = 0.434)(Fig. 3a) or T0 to T2 (${\chi }_{2}^{2}$ = 3.79, *p* = 0.151) (Fig. 3b). Compared to parents who had not received results by their T2 time point, those who had received results had higher satisfaction scores (satisfaction deficit ratio=0.56, 95% CI = [0.23, 1.38], p = 0.21), after adjusting for T0 satisfaction (Fig. 3b). Time taken to receive results was not associated with changes in satisfaction ratings from T0 to T1 (deficit ratio = 1.00, 95% CI = [1.00, 1.01], ${\chi }_{1}^{2}$ = 1.08, *p* = 0.298), nor was the type of GGS result received at T1 (${\chi }_{2}^{2}$ = 2.36, *p* = 0.307), adjusting for T0 satisfaction and time taken to receive results (Fig. 3a).

#### Recommending PREDICT to others

At T0, most children were willing to recommend participating in PREDICT to others (12/18, 67%). The remainder were unsure (6/18, 33%). At T1, most children remained willing to recommend PREDICT to others (6/10, 60%) or were unsure (4/10, 40%). We did not identify any evidence to suggest these responses changed over time (*p* = 0.119).

Most parents reported that they would recommend PREDICT to others at T0 (154/175, 88%). A small proportion would not (1/175, 1%) or were unsure (20/175, 11%). At T1, 72/75 (96%) of parents were willing to recommend PREDICT to others with the remainder unsure (3/75, 4%). At T2, 79/90 (88%) would recommend, with the remainder still unsure (11/90, 12%). We found no evidence to suggest parent responses changed between T0 and T1 (*p* = 0.401), and T0 and T2 (*p* = 1.000).

#### Parent regret

**Fig. 4:**
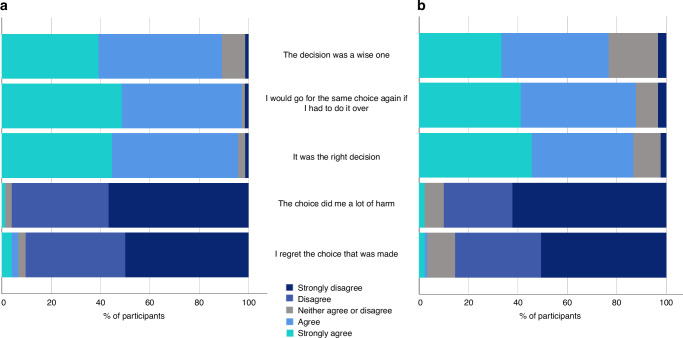
Parent responses to five items on the Decisional Regret Scale. **a** Post-results (T1) and **b** 1-year post-enrolment (T2). At T1, *n* = 74 for all items except ‘I would go for the same choice again if I had to do it over, where *n* = 72. At T2, *n* = 90 for all items except ‘I regret the choice that was made’ where *n*=89.

At T1, regret was low with a mean score of 15.74 (SD = 15.72) and 24/74 (32%) of parents scoring the minimum score of 0. At T2, regret remained low with a mean score of 18.45 (SD = 18.84) and 25/90 (28%) scoring 0. At both timepoints, most parents did not regret their choice or believe it caused them harm (Fig. 4).

#### Qualitative themes characterising overall satisfaction

Most participants qualitatively expressed satisfaction with their participation in PREDICT gained through finding purpose and emotional relief or reassurance. However, some described systemic or procedural challenges that compromised their trust in the study, limiting satisfaction (Table 2).

#### Finding purpose and personal benefit

The opportunity to contribute to research and help their family or other families through their participation was a common source of satisfaction described by parents and children. Parents also described being satisfied because their participation provided emotional relief, even if there was a reportable finding, as it provided more information and enabled proactive choices.

#### Compromised trust in the system and process

Participants’ dissatisfaction stemmed from challenges in communication, trust, and procedural logistics, which shaped overall experiences of the study. Poor communication eroded trust, with fragmented or unclear communication at the consent or result disclosure stage leaving some parents feeling anxious, and even distressed, due to “not getting any results of the tests that were done” (mother, 45 yrs). Concerns about privacy and data-sharing also weakened confidence in the study’s procedural integrity for those who had “concerns over how personal medical information is stored” (father, 39 years). Logistical challenges, particularly the gap between receiving results and attending a genetic appointment, added practical strain while also undermining parents’ trust in study procedures. These delays were described as an added source of frustration and dissatisfaction, with genetics appointments described by one parent as “practical, comforting, [and] realistic” (mother 45 years), and wishing they could have happened sooner.

## Discussion

We contribute novel insights into families’ experiences with standalone trio-GGS for childhood cancer in a cohort of unselected, newly diagnosed patients. Using a multi-perspective, mixed-methods, longitudinal approach, we found that whilst parents and children reported low-to-moderate levels of consent-related and result-related distress and high overall satisfaction with their participation, some reported experiencing challenges at consent, result disclosure, and up to one-year post-enrolment. Together, the quantitative and qualitative data reveal the emotional and practical complexities of undergoing trio testing at cancer diagnosis. As broad GGS in childhood cancer moves beyond the research space and towards implementation in standard clinical practice, our findings point to key opportunities to inform how this testing is delivered, communicated, and supported to enhance families’ experiences.

Less than half of parents and only one child reported thoroughly reading consent materials, consistent with previous findings in a precision medicine study for high-risk cancer [32]. Our qualitative data suggested this was due to overwhelm related to the child’s recent cancer diagnosis, in line with prior literature on consent for paediatric cancer research [8, 41–43]. Further, trust in clinicians or health systems led some parents and children to assume participation was in their best interest, rather than a result of having critically engaged with study information. Whilst this deferral of responsibility may serve to protect families from emotional overwhelm, it limits capacity for informed consent. In paediatric oncology, blurred lines between research and clinical care can lead families to trust research recommendations as though they are essential treatment decisions, complicating consent [42, 44, 45]. These findings suggest consent processes at diagnosis should be flexible, responsive to families’ emotional needs, and potentially revisited to ensure truly informed participation.

Additionally, parents qualitatively described a strong desire to do ‘whatever it takes’ for their child, which may have impaired their ability to consider implications of participation for themselves. Sequencing parent genomes as part of trio-GGS introduces ethical tensions: parental distress and urgency to benefit their child may compromise capacity for informed decision-making for themselves. Given trio-based studies can potentially diagnose parents with a genetic cancer risk despite the primary study’s focus on the child, it is important that parents have carefully considered potential consequences of participation for themselves. As broad GGS moves from research into clinical use, our findings suggest that a flexible, staged approach to testing, such as initial singleton sequencing with parental testing as needed, may better support informed consent for testing by reducing complexity and maintaining focus on the child. Alternatively, strategies aimed at improving parent consent comprehension, such as online information and consent modules, could be considered.

Although most parents and children reported low consent-related distress, qualitative data revealed emotional and practical challenges. For some parents, the prospect of discovering they had passed on a cancer-predisposing variant to their child was distressing. Our quantitative measure assessing parental consent-related distress focused on consent processes (e.g. providing a sample) and may not have captured this kind of emotional burden, often referred to as transmission guilt, which is well documented in the literature [8, 10, 12]. Parents also described logistical challenges associated with parental sample collection, echoing issues reported by clinicians delivering this study [22]. These logistical difficulties may not have registered as ‘distress’ in a conventional sense in quantitative assessments. This divergence between quantitative and qualitative findings underscores the need for clinical approaches that go beyond surface-level assessments of distress. Even when standard measures suggest low distress, families may still benefit from access to embedded genetic counselling and psychological support, as well as flexible, tailored consent processes that recognise and respond to hidden emotional and logistical burdens [43]. This is particularly important to address the needs of those identified as more vulnerable to consent-related distress, which our findings suggest includes parents with lower household incomes or below average self-reported genetics knowledge.

Distress levels at result-return were moderate for both parents and children. Importantly, we found no evidence of a difference in parents’ result-related distress between those who received reportable findings, a recommendation for a clinical genetics referral, or no findings. This contrasts findings from a recent US-based study, which reported parents of children participating in paired tumour-germline sequencing experience greater distress at result disclosure if their child received a P/LPV, compared to those without [25], and that parents with P/LPV or uncertain results may require explicit screening and psychosocial support interventions [13]. While this discrepancy may reflect differences in testing contexts, study methodologies, patient populations, or healthcare systems between the US and Australia, our qualitative data supported our findings. Qualitative descriptions of parental emotional reactions to results varied from positive experiences, such as relief and reassurance, to negative ones, such as disappointment or distress. Results did not predict parents’ emotional response, with the full range of reactions described by those with and without reportable findings. Whilst current research and clinical processes typically prioritise return of informative results, our data emphasise the value of returning all results, including uninformative ones, to ensure families receive the necessary support and can benefit from emotional relief.

Overall, most parents and children were satisfied with their participation in PREDICT, would recommend participating to others, and parents reported low regret. Parents and children qualitatively described experiencing satisfaction with their participation through making altruistic contributions to research and gaining emotional relief through receiving results. While this aligns with previous studies examining satisfaction with paired and germline-only genomic sequencing in childhood cancer [10, 33], a substantial proportion of children were unsure whether they would recommend participating in PREDICT. This may reflect limited understanding among children of PREDICT’s purpose and implications, which aligns with their low engagement with consent information. Additional efforts are needed to support child understanding and engagement with testing information. This would ensure children can derive meaning or benefit from participation, and reduce negative psychosocial impacts associated with confusion or unmet information needs [8].

Despite high overall satisfaction scores, qualitative data revealed that sources of frustration existed, presenting opportunities to improve GGS delivery. Communication and trust emerged as important factors influencing parental satisfaction, shaping experiences during both consent and result-return. Unclear communication at these timepoints was linked to reported negative experiences and eroded trust in the study and testing process. However, a notable paradox emerged: while some parents valued receiving results from trusted, familiar clinicians, such as their child’s treating oncologist, others were frustrated by those clinicians’ self-described limited genetics expertise. Delays in result-return or access to genetic counselling also left parents feeling distressed or unsupported, with counselling appointments described as alleviating anxiety. Whilst the study genetic counsellor was available to provide additional support to clinicians or families pre or port result delivery, this was not compulsory or routinely implemented [29]. Similar communication challenges, particularly around result-return, have been reported in other studies of paired tumour-germline sequencing in children with high-risk cancers, prompting calls for improved communication and better access to genetic counselling [17, 33]. Clinically embedding and properly utilising genetic counsellors early in the GGS process could help build trust and improve communication by supporting timely result delivery, improving recall and understanding of results, and providing psychosocial support [46].

The unselected cohort was a key strength of PREDICT. Our sample was representative of PREDICT families across key clinical and demographic characteristics, including the child’s cancer diagnosis, enhancing the generalisability of our findings. Incorporating multiple perspectives, particularly that of child participants, further strengthened our study. However, despite targeted efforts, our sample was predominantly mothers, and the small patient sample impeded meaningful statistical analyses for this group. Given fathers and children have been identified as priority populations in psychosocial oncology research [47, 48], future studies should prioritise their recruitment. Exclusion of non-English speaking families reduced cultural and linguistic diversity, and self-selection bias may have favoured families who are more supportive of research. Attrition and variation in the timing of questionnaires and follow-up may also have influenced responses. While validated measures were used where possible, distress and satisfaction are complex and may have been shaped by factors not explored in this study. Future studies should attempt to assess distress using validated measures with established clinical cut-offs. Although no evidence of a statistical association was observed between parent distress or satisfaction and receiving a reportable germline finding, the sample size relative to the number of predictors may have limited statistical power. The sample size was also insufficient to explore differences by different types of reportable findings. Results indicating a childhood-onset cancer predisposition may, for example, elicit different reactions than a carrier result for a recessive cancer predisposition gene. Further, when including family history of cancer as a predictor in our mixed models we chose to include participants whose family history information was ‘not provided’ as a level rather than treat it as missing to preserve sample size. These findings should therefore be interpreted with caution as we were unable to distinguish within this level between those who had no family history of cancer, those unaware of their family history, and those who chose not to disclose.

Our study identified several key considerations for the integration of GGS for genetic cancer risk into routine childhood cancer care. Families were mostly satisfied with their participation and did not experience high levels of consent or result-related distress. However, some reported emotional and practical challenges during the consent process, suggesting a need for more flexible, tailored approaches that reflect individual needs and preferences. This may involve delaying the approach for germline consent for families experiencing distress or overwhelm related to the child’s cancer diagnosis, or revisiting germline consent preferences once families have had time to adjust to the cancer diagnosis. Trio testing also introduced challenges for some families, particularly around the time of cancer diagnosis when parents may be distressed and focused on their child’s immediate treatment. As a result, they may not fully consider testing implications for themselves. Future trio-based studies should account for this or consider alternative approaches if trio testing is not clinically urgent. Clear, timely communication from trusted healthcare professionals was important to families, especially regarding result-return. Additionally, given the range of emotional responses to receiving results, consistent return of findings, regardless of result type, is important and may support early identification of families who could benefit from psychosocial support. Embedding genetic counsellors early in the GGS process could help improve communication and further exploring barriers to genetic counsellor integration in mainstream GGS should be considered.

## Supplementary information

Supplementary Material

## Data Availability

De-identified participant data and the PREDICT-Impact data dictionary can be made available on request to the corresponding author.
