# Supplementary material for: “I don’t think I even thought of myself” A mixed-methods study of family experiences of trio germline whole genome sequencing in newly diagnosed childhood cancer
Source: Br J Cancer. 2026 Mar 5;134(9):1336–51. doi: 10.1038/s41416-026-03354-9 (PMC13079812; doi:10.1038/s41416-026-03354-9)
Supplement: Supplementary file 1 — Supplementary Material [file 41416_2026_3354_MOESM1_ESM.docx]

**Supplementary Table 1. Questionnaire measures completed by parents at baseline/post-consent (T0), after the return of PREDICT results (T1), one year after enrolment (T2) and data collected from the study database (SDB)**

| **Aim** | **Item** | **Measure and response option** | **T0** | **T1** | **T2** | **SDB** |
| --- | --- | --- | --- | --- | --- | --- |
| **Demographics** | - Age  - Sex  - Whether they have other children (and their ages)  - Education  - Employment status  - Health and life insurance status  - Additional financial support  - Annual household income  - Marital status  - Cultural background  - Religious background  - Self-rated genetics knowledge  - Sample provided (trio) | mixture of open-ended (e.g. postcode) and forced choice options (e.g. employment status) depending on the question  some basic demographics collected from PREDICT study database | X | - | - | X |
| **Demographics and clinical information of child** | - Child sex  - Child age at cancer diagnosis  - Child age at enrolment on PREDICT  - Time from cancer diagnosis to enrolment  - Cultural background  - Geographic location (postcode)  - Cancer diagnosis  - PREDICT result (findings, time to receipt)  - Family history of cancer | data extracted from PREDICT study database for all children of participating parents | - | - | - | X |
| **Experience of consent** | When you were invited to participate in the PREDICT study, you would have received an information sheet and consent form about the study. Please indicate whether you read the information: | ‘from cover to cover,’ ‘quite thoroughly,’ ‘just the parts I felt were relevant,’ ‘briefly,’ ‘I didn’t read the information sheet because: (open-ended).’ | X |  |  |  |
|  | The length of the information sheet and consent form was: | ‘too long,’ ‘just right,’ ‘too short’ | X |  |  |  |
|  | How distressing for you was…   - Your child’s cancer diagnosis? - Deciding whether to provide a sample of *your child's* DNA to look for gene faults linked to cancer predisposition syndromes? - Deciding whether to provide a sample of *your* DNA to look for gene faults linked to cancer predisposition syndromes?   Please provide further details of any distress you experienced related to the genetic testing process: open-ended | scale from 1-10 where 1= ‘not at all’ and 10= ‘extremely’ | X | X |  |  |
| **Experience of result return** | How distressing for you was…   - Waiting for y*our child's* genetic test results? - Receiving your child's genetic test results? | scale from 1-10 where 1= ‘not at all’ and 10= ‘extremely’ or ‘N/A – I have not received my child’s genetic test result’ |  | X |  |  |
|  | Did your child's doctor ask you to provide a sample of your DNA to look for gene faults linked to cancer predisposition syndromes? | ‘yes,’ ’no’ |  | X |  |  |
|  | If yes to above, how distressing for you was…   - Waiting for your genetic test results? - Receiving your genetic test results?   Please provide further details of any distress you experienced related to the genetic testing process: open-ended | scale from 1-10 where 1= ‘not at all’ and 10= ‘extremely’ or ‘N/A – I have not receive my genetic test result’ |  | X |  |  |
| **Overall satisfaction with PREDICT participation** | At the moment, how satisfied are you with your decision about being part of PREDICT? If you are not satisfied, please tell us why: open-ended | scale from 0 to 100 where 0= ‘not at all’ and 100= ‘completely’ | X | X | X | - |
|  | Would you recommend being involved in the PREDICT study to another parent/patient?  If no/unsure, please tell us why: open-ended | ‘yes,’ ‘no,’ ‘unsure’ | X | X | X |  |
|  | Thinking about the decision you made about participating in the PREICT study   1. It was the right decision 2. I regret the choice that was made 3. I would go for the same choice if I had to do it over again 4. The choice did me a lot of harm 5. The decision was a wise one | ‘strongly disagree,’ ‘disagree,’ ‘neither agree nor disagree,’ ‘agree,’ ‘strongly agree’ (Decision Regret Scale, Cronbach's alpha = 0.81 to 0.92^32^)  To produce a global score using the Decision Regret Scale, participant responses to each 5-point Likert Scale item (1 = strongly agree to 5 = strongly disagree) are converted to a 0–100 scale by subtracting 1 and multiplying by 25. Scores for items 2 and 4 are reverse-coded so that higher scores consistently indicate greater regret. The global score is then calculated by summing all five converted items and dividing the total by 5. Final scores range from 0 (no regret) to 100 (high regret), increasing in increments of 5. | - | X | X | - |

**Supplementary Table 2. Questionnaire measures completed by children at baseline/post consent (T0) and after return of PREDICT results (T1) and data collected from the study database (SDB)**

| **Aim** | **Item** | **Measure and response option** | **T0** | **T1** | **SDB** |
| --- | --- | --- | --- | --- | --- |
| **Demographics and clinical information** | - Child sex  - Child age at cancer diagnosis  - Child age at enrolment on PREDICT  - Time from cancer diagnosis to enrolment  - Cultural background  - Cancer diagnosis  - Geographic location (postcode)  - PREDICT result (findings, when received, time to receipt) | Data extracted from PREDICT study database for all participating children | - | - | X |
| **Experience of consent** | At this stage, how do you feel about being part of the PREDICT study? | (open-ended) | X |  |  |
|  | When you first heard about the PREDICT study, what were your thoughts about it? (e.g. you were happy or worried?). There is no right answer. Please tell us whatever you thought: | (open-ended) | X |  |  |
|  | When you were invited to participate in the PREDICT study, you would have received an information sheet and consent form about the study. Please indicate whether you read the information: | ‘from cover to cover,’ ‘quite thoroughly,’ ‘just the parts I felt were relevant,’ ‘briefly,’ ‘I didn’t read the information sheet because: (open-ended)’ | X | - | - |
|  | The length of the information sheet and consent form was: | ‘too long,’ ‘just right,’ ‘too short’ | X | - | - |
|  | Has being a part of the PREDICT study so far made you feel worried at all?  What made you feel worried (if at all): open-ended | “Not at all”, “a little”, “somewhat”, “quite a bit”, “very much” | X | - | - |
|  | Has being a part of the PREDICT Study made you feel happy (or reassured) at all?  What made you feel reassured (if at all): open-ended |  |  |  |  |
| **Experience of return of result** | Has being a part of the PREDICT study made you feel worried at all?  What made you feel reassured (if at all): open-ended | “Not at all”, “a little”, “somewhat”, “quite a bit”, “very much” |  | X | - |
|  | Has being a part of the PREDICT Study made you feel happy (or reassured) at all?  What made you feel reassured (if at all): open-ended |  |  |  |  |
| **Overall Satisfaction of PREDICT participation** | At the moment, how satisfied are you with your decision about being part of PREDICT?  If you are not satisfied, please tell us why: open-ended | 0 (“not at all”) to 100 (“completely”) | X | X | - |
|  | Would you recommend being involved in the PREDICT study to other people? | ‘yes,’ ‘no,’ ‘unsure’ | X | X | - |

| Supplementary Table 3. Parents’ post-results (T1) semi-structured interview guide | |
| --- | --- |
| Topic | **Questions and prompts** |
| Introduction | “Sometimes telling your story may bring up a range of feelings or memories. So, just before we get started I wanted to check in to see how you are feeling. On a scale of 0-10 where 0 is “None” and 10 is “Extreme” how much distress have you experienced this past week, including today?” (followed by anxiety, depression, anger and need for help) [if >8, see risk management protocol at the end of the interview schedule]  “Do you have any questions? Please let me know if you are feeling distressed or would like to stop at any point.”  “Just to get to know you a little, could you tell me briefly a little about your child and their experience with cancer?’ |
| Consent experiences | “Now I’d like to talk a little about your understanding of the PREDICT study that you and child have enrolled in?”  “Who first approached you about becoming involved in the ‘study?”  “How did you feel about it at the time?”   - - Probe: “How did you feel about the timing of being invited to the study?”   “What were your reasons for enrolling your child and yourself in the study?”   - - Probe: “What benefits did you expect for your child?”   - Probe: “Did you have any concerns for your child?”   - Probe: “Did you have any concerns for yourself?”   “What did you think about the amount of information given in the Information Sheet to you about what was being done in the study?”   - - Probe: “Who gave the information? Would you have preferred to receive the information in a different way/at a different time? Who did you find helpful to discuss this study with before deciding to participate? Have you spoken to any other parent enrolled in the study?”   “In regard to providing a blood sample for the stud, how did you feel about providing your child’s sample?”   - - Probe (if relevant): “How did you feel about providing your own blood sample? (i.e. trio testing)” |
| Result experiences | “I’m now going to ask you about your reaction to the results of the study (if any). You can tell me about your reactions without telling me your child’s actual results, if you would prefer. Have you been told your child was born with a ‘gene fault’ that may have contributed contribute to the development of cancer?”   - - If yes: “Can you tell me what that information means for your child?”   - Probe if relevant: “Have you been told that the ‘gene fault’ was inherited?”   - Probe if yes: “Do you know which parent passed the gene fault to your child?”   “How did you emotionally react to that information?”  “How did you receive the results? Would you have preferred to receive them in another way?”  “Did you feel comfortable asking questions about what the results meant?”   - - Probe if yes: “Tell me about the environment that made you feel comfortable asking questions?”   - Probe if no: “Tell me about the environment that made you feel uncomfortable asking questions?”   “Do you remember how long it took to get a result from the study? What was this wait like for you?”   - - Probe if wait was difficult: “What strategies did you use to cope with the difficult waiting time?” |
| Overall satisfaction | “Were you satisfied with the amount of information you received about the results of testing of your child’s cancer-related genes? If not, why?”   - - Probe if relevant: “Were you satisfied with the amount of information you received about the results of testing in you? If not, why?”   - Probe: “Was there anything in particular that helped you understand this information?”   - Probe: “Would you have wanted more or less information?”   - Probe: “Did you find this information useful in helping understand why your child developed cancer?”   “Now that you have been through the process, was there anything your child’s medical team could have improved?”  “If you could go back in time, would you have changed your decision to be involved in the ‘Cancer Predisposition’ study? Why/why not?” |
| Closing | “Just to finish up I just wanted to ask you to rate how you are feeling for me, just like as did at the start of the interview. On a scale of 0-10 where 0 is “None” and 10 is “Extreme”, how much DISTRESS are you feeling now? (followed by anxiety, depression, anger and need for help) ***[If >8, go to risk management protocol]*** Okay, thank you so much for that. Before I go do you have any final questions or concerns at all?” |

**Supplementary Table 4. Results of ordinal mixed model analysis of parent distress related to the consent process at baseline/post-consent (T0) and key demographic and clinical predictors.**

|  | **Distress related to providing child’s DNA for CPG analysis (T0)** | | | | | **Distress related to providing own DNA for CPG analysis (T0)** | | | | |
| --- | --- | --- | --- | --- | --- | --- | --- | --- | --- | --- |
| Predictor | OR | 95% CI | z | p | 𝑥(df) | OR | 95% CI | z | p | 𝑥(df) |
| Marital status (not married/defacto vs married/defacto) | 1.37 | 0.50, 3.76 |  | 0.542 | 0.37(1) | 1.30 | 0.39, 4.35 |  | 0.670 | 0.18(1) |
| Family history: |  |  |  | 0.888 | 0.24(2) |  |  |  | 0.525 | 1.29(2) |
| Yes vs no | 1.02 | 0.33, 3.17 | 0.03 | 0.978 |  | 1.15 | 0.27, 4.83 | 0.19 | 0.853 |  |
| Not provided vs no | 1.20 | 0.56, 2.58 | 0.48 | 0.635 |  | 1.74 | 0.66, 4.58 | 1.12 | 0.261 |  |
| Other children (no vs yes) | 1.47 | 0.54, 4.05 | 0.75 | 0.453 | 0.56(1) | 1.86 | 0.52, 6.61 |  | 0.338 | 0.92(1) |
| Income |  |  |  | **0.003** | 11.95(2) |  |  |  | 0.107 | 4.47(2) |
| >$120k vs <$120k | 0.23 | 0.09, 0.57 | −3.20 | 0.001 |  | 0.33 | 0.12, 0.95 | -2.05 | 0.041 |  |
| Prefer not to answer vs <$120k | 1.00 | 0.38, 2.61 | −0.01 | 0.992 |  | 0.81 | 0.26, 2.57 | -0.36 | 0.720 |  |
| Genetics knowledge (below average vs average and above) | 2.29 | 1.10, 4.76 |  | **0.027** | 4.87(1) | 3.22 | 1.31, 7.90 |  | **0.011** | 6.54(1) |
| Time from cancer diagnosis to enrolment | 1.00 | 1.00, 1.01 |  | 0.496 | 0.46(1) | 1.00 | 1.00, 1.011 |  | 0.372 | 0.78(1) |
| Cancer diagnosis |  |  |  | 0.096 | 4.68(2) |  |  |  | 0.093 | 4.76(2) |
| CNS vs haematological | 0.37 | 0.10, 1.44 | −1.44 | 0.151 |  | 0.26 | 0.05, 1.46 | -1.53 | 0.126 |  |
| Non-CNS solid vs haematological | 0.47 | 0.21, 1.03 | −1.88 | 0.061 |  | 0.38 | 0.14, 1.05 | -1.86 | 0.063 |  |
| Parent gender (female vs male) | 1.44 | 0.76, 2.74 |  | 0.264 | 1.25(1) | 1.46 | 0.72, 2.99 |  | 0.299 | 1.08(1) |
| CPG=cancer predisposition gene  CNS=central nervous system | | | | | | | | | | |

| **Supplementary Table 5. Results of linear mixed model analysis of parent distress related to receiving their child’s genetic results at result-return (T1) and key demographic and clinical predictors.** | | | | | |
| --- | --- | --- | --- | --- | --- |
| Predictor | Estimate | 95% CI | t | p | F(df1, df2) |
| Marital status (not married/defacto vs married/defacto) | 1.24 | -1.12, 3.59 |  | .295 | 1.12(1, 54) |
| Family history: |  |  |  | 0.177 | 1.79(2, 54) |
| Yes vs no | -0.53 | -2.75, 1.69 | -0.48 | .635 |  |
| Not provided vs no | -1.28 | -2.66, 0.10 | -1.86 | .068 |  |
| Other children (no vs yes) | -1.64 | -3.74, 0.46 |  | .124 | 2.45(1, 54) |
| Income |  |  |  | 0.612 | 0.50(2, 54) |
| >$120k vs <$120k | 0.16 | -1.52, 1.84 | 0.19 | .847 |  |
| Prefer not to answer vs <$120k | -0.52 | -2.10, 1.06 | -0.66 | .513 |  |
| Genetics knowledge (below average vs average and above) | 0.50 | -0.94, 1.94 |  | .489 | 0.49(1, 54) |
| Cancer diagnosis |  |  |  | 0.637 | 0.46(2, 54) |
| CNS vs haematological | -0.32 | -1.76, 1.11 | -0.45 | .652 |  |
| Non-CNS solid vs haematological | 0.47 | -1.53, 2.48 | 0.47 | .637 |  |
| Parent gender (female vs male) | -0.91 | -2.16, 0.33 |  | .145 | 2.22(1,34.17) |
| Time from enrolment to results | -0.00 | -0.01, 0.01 |  | .716 | 0.13(1, 51.30) |
| PREDICT result |  |  |  | 0.118 | 2.23(2, 54) |
| Reportable finding vs no findings | 1.68 | 0.08, 3.28 | 2.11 | .040 |  |
| Clinical genetics referral vs no findings | 0.78 | -1.10, 2.66 | 0.83 | .409 |  |
| CNS=central nervous system | | | | | |

| **Supplementary Table 6. Demographics of parents participating in PREDICT-Impact.** | | |
| --- | --- | --- |
|  | **All participating parents (N=187)** | **Interview participants (N=49)** |
| **Age, years** |  |  |
| Mean (SD) | 40.4 (7.0) | 39.9 (8.1) |
| Range | 21.8-59.3 | 21.8-55.9 |
| (missing) | 31 | 5 |
| **Gender, no. (%)** |  |  |
| Female (mothers ^a^) | 116 (62.0) | 37 (75.5) |
| Male (fathers) | 71 (38.0) | 12 (24.5) |
| **Highest level of education, no. (%)** |  |  |
| High school only | 27 (14.4) | 8 (16.3) |
| Post-high school (inc. vocational training) | 161 (85.6) | 41 (83.7) |
| **Employment, no. (%)** |  |  |
| Employed: Full-time | 96 (51.3) | 22 (44.9) |
| Employed: Part-time/casual | 54 (28.9) | 19 (38.8) |
| Not employed: Actively seeking work | 3 (1.6) | - |
| Not employed: Not seeking work/retired/student | 10 (5.3) | 2 (4.1) |
| Not employed: Home duties | 21 (11.2) | 6 (12.2) |
| (missing) | 3 (1.6) | - |
| **Insurance, no. (%)** |  |  |
| Private health insurance | 76 (40.6) | 20 (41.7) |
| Life insurance | 5 (2.7) | - |
| Private health and life insurance | 48 (25.7) | 16 (33.3) |
| Medicare only (public healthcare) | 58 (31.0) | 12 (25.0) |
| **Marital status, no. (%)** |  |  |
| Never married/never de facto | 7 (3.7) | 3 (6.1) |
| Currently married or de facto | 159 (85.0) | 42 (85.7) |
| Separated/divorced/previous de facto/widowed | 19 (10.2) | 3 (6.1) |
| (missing) | 2 (1.1) | 1 (2.0) |
| **Aboriginal or Torres Strait Islander, no. (%)** | |  |
| Yes | 10 (5.3) | - |
| No | 175 (93.6) | 48 (98.0) |
| (missing) | 2 (1.1) | 1 (2.0) |
| **Cultural background, no. (%)** |  |  |
| Australian | 132 (70.6) | 40 (81.6) |
| Western/European | 15 (8.0) | 3 (6.1) |
| Non-Western/European | 36 (19.3) | 6 (12.2) |
| Missing | 4 (2.1) | - |
| **Preferred language, no. (%)** |  |  |
| English | 175 (93.6) | 47 (95.9) |
| Other | 8 (4.3) | 2 (4.1) |
| Missing | 4 (2.1) | - |
| **Religion, no. (%)** |  |  |
| No religion | 76 (40.6) | 22 (44.9) |
| Christianity | 78 (41.7) | 20 (40.8) |
| Islam | 11 (5.9) | 1 (2.0) |
| Buddhism | 3 (1.6) | 1 (2.0) |
| Hinduism | 9 (4.8) | 1 (2.0) |
| Judaism | 4 (2.1) | 2 (4.1) |
| Catholic | 6 (3.2) | 2 (4.1) |
| **Household income, no. (%)** |  |  |
| Less than $29,999 | 16 (8.6) | 5 (10.2) |
| $30,000-$59,999 | 21 (11.2) | 5 (10.2) |
| $60,000-$89,999 | 19 (10.2) | 2 (4.1) |
| $90,000-$119,999 | 20 (10.7) | 3 (6.1) |
| $120,000 and above | 77 (41.2) | 23 (46.9) |
| Prefer not to answer | 33 (17.6) | 11 (22.4) |
| (missing) | 1 (.5) |  |
| **Receiving additional financial support, no. (%)** |  |  |
| No | 122 (65.2) | 27 (55.1) |
| Government/organisation | 34 (18.2) | 11 (22.4) |
| Parent, spouse or partner | 30 (16.0) | 11 (22.4) |
| (missing) | 1 (0.5) | - |
| **Number of other children, no. (%)** |  |  |
| 0 | 29 (15.5) | 7 (14.3) |
| 1 | 96 (51.3) | 24 (49.0) |
| 2 | 46 (24.6) | 15 (30.6) |
| 3+ | 12 (6.4) | 1 (2.0) |
| (missing) | 4 (2.1) | 2 (4.1) |
| **Perceived genetics knowledge, no. (%)** |  |  |
| Below average | 68 (36.4) | 18 (36.7) |
| About average | 101 (54.0) | 29 (59.2) |
| Above average | 14 (7.5) | 1 (2.0) |
| (missing) | 4 (2.1) | 1. (2.0) |
| a. One participating mother was the birth mother but not the biological mother; her child was conceived child via a donor egg from a biological sister. | | |
